# MYH9 facilitates autoregulation of adipose tissue depot development

**DOI:** 10.1172/jci.insight.136233

**Published:** 2021-05-10

**Authors:** Sin Ying Cheung, Mohd Sayeed, Krishnamurthy Nakuluri, Liang Li, Brian J. Feldman

**Affiliations:** 1 Department of Pediatrics,; 2 Nutrition Obesity Research Center,; 3 Diabetes Research Center, and; 4 Developmental and Stem Cell Biology Program, University of California, San Francisco, San Francisco, California, USA.

**Keywords:** Development, Endocrinology, Adipose tissue, Adult stem cells

## Abstract

White adipose tissue not only serves as a reservoir for energy storage but also secretes a variety of hormonal signals and modulates systemic metabolism. A substantial amount of adipose tissue develops in early postnatal life, providing exceptional access to the formation of this important tissue. Although a number of factors have been identified that can modulate the differentiation of progenitor cells into mature adipocytes in cell-autonomous assays, it remains unclear which are connected to physiological extracellular inputs and are most relevant to tissue formation in vivo. Here, we elucidate that mature adipocytes themselves signal to adipose depot–resident progenitor cells to direct depot formation in early postnatal life and gate adipogenesis when the tissue matures. Our studies revealed that as the adipose depot matures, a signal generated in mature adipocytes is produced, converges on progenitor cells to regulate the cytoskeletal protein MYH9, and attenuates the rate of adipogenesis in vivo.

## Graphical abstract

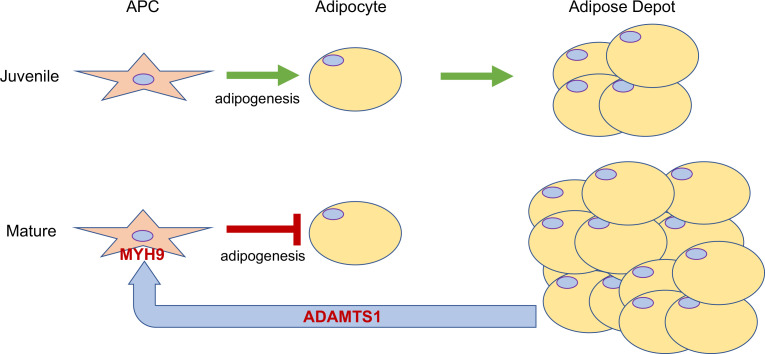

## Introduction

White adipose tissue (WAT) is a dynamic tissue that influences a variety of important physiological processes, including energy storage and insulin signaling, and secretes a number of endocrine and paracrine factors with broad effects (1–4). A substantial proportion of WAT forms postnatally by the differentiation of adipose depot–resident precursor cells (APCs) in a process called adipogenesis. Adipogenesis has been heavily studied using reductionist in vitro and tissue culture model systems. These studies identified a number of factors that can modulate adipogenesis within the context of cell-autonomous in vitro assays (5–7). However, advantages of the postnatal timing of the development of adipose tissue have not been fully leveraged to elucidate the in vivo mechanisms that regulate this important developmental process. Further, importantly, the relevant intracellular pathways and mechanisms in APCs that respond to extracellular signals and regulate adipogenesis have not been fully defined.

We previously identified ADAMTS1 as an extracellular signaling factor with levels that respond to changes in systemic hormone levels as well as to the number of calories consumed in mice and humans (8). We revealed that when ADAMTS1 is secreted from mature adipocytes in WAT, it can transmit a signal to WAT depot–resident APCs (8). Here, we describe our studies examining whether an adipocyte-derived extracellular signal has a role in the physiological process of adipose depot formation and, if so, what the mechanism of this regulation is.

We discovered that nonmuscle myosin protein, MYH9 (also called nonmuscle myosin heavy chain 2A) levels were dynamic during adipose depot development and this shift, transmitted by the extracellular ADAMTS1-Wnt signal, modulated adipogenesis in APCs. Further, we found that this pathway was deployed during adipose depot development during the critical transition window when depots are undergoing substantial expansion to when depots are largely mature and at homeostatic adult size.

## Results

### ADAMTS1 levels in WAT increase when mice reach 8 weeks of age.

**Figure 1:**
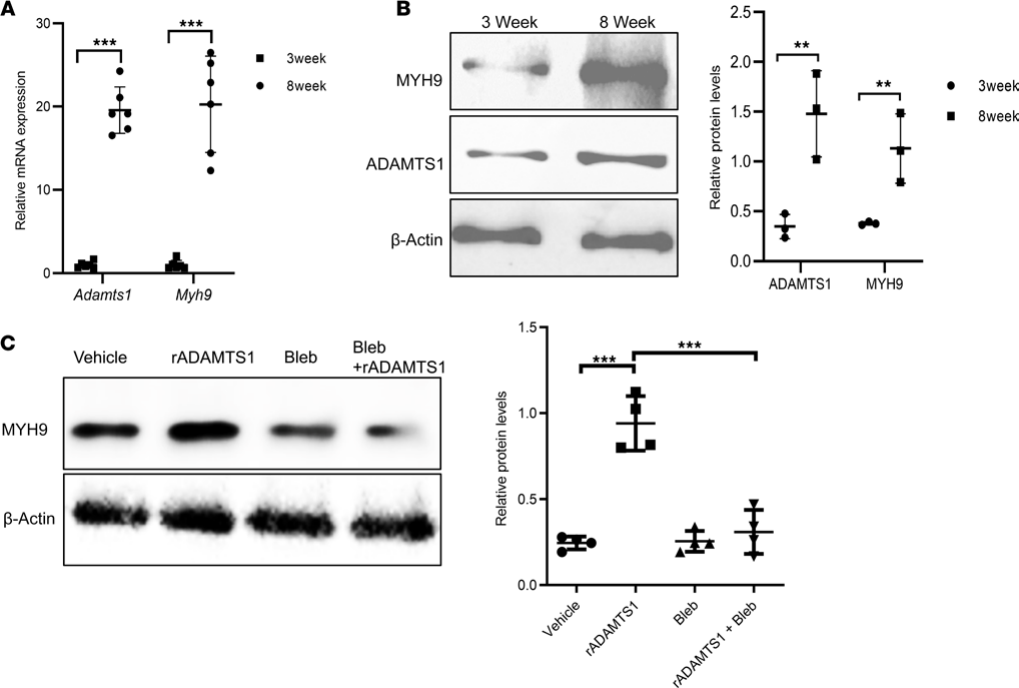
Adamts1 and Myh9 expression changes during adipose depot development. (**A**) RT-qPCR quantifying *Adamts1* and *Myh9* expression levels in white adipose tissue harvested from 3-week-old and 8-week-old male wild-type mice (*n* = 6). (**B**) Immunoblots (left) with quantification (right) of ADAMTS1 and MHY9 from white adipose tissue harvested from 3-week-old and 8-week-old male wild-type mice and their quantification (*n* = 3). (**C**) Immunoblots (left) with quantification (right) of MYH9 levels in APCs isolated from male wild-type mice and treated with rADAMTS1 (100 ng/mL), myosin II inhibitor blebbistatin (Bleb) (30 μM), or both rADAMTS1 and Bleb (*n* = 4). Error bars represent mean ± SD. *P* values were calculated using *t* tests. **P < 0.01, ***P < 0.001. Post hoc Bonferroni’s test was performed in **C**.

**Figure 2:**
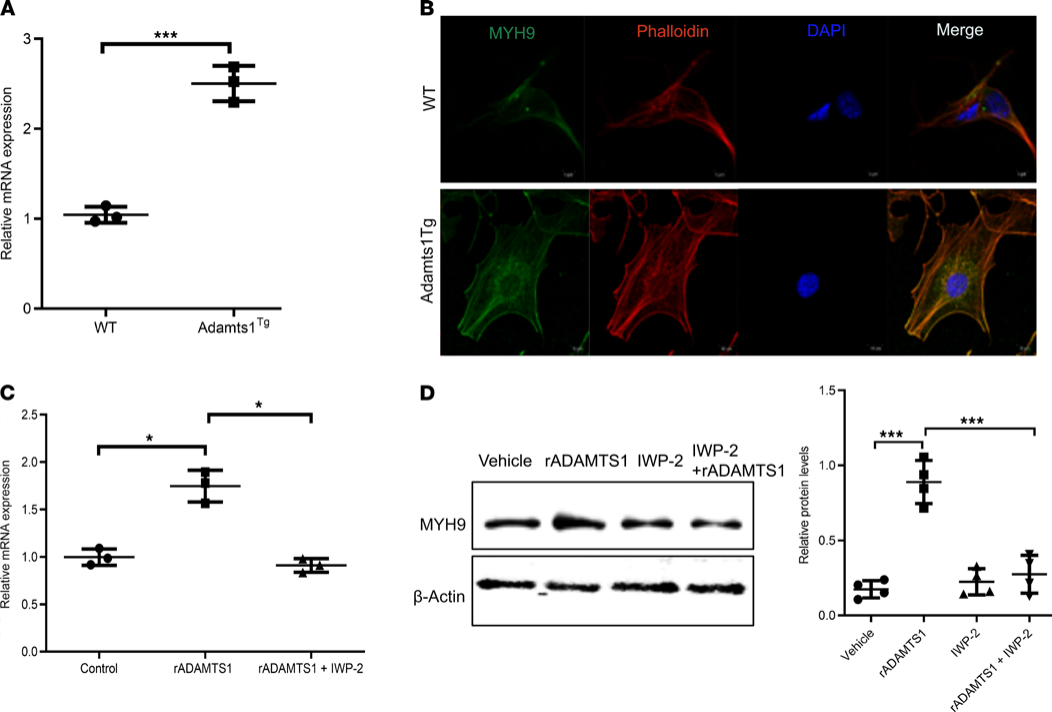
ADAMTS1 regulates MYH9 in APCs. (**A**) RT-qPCR quantifying *Myh9* expression levels in APCs isolated from subcutaneous white adipose tissue depots from 3-week-old male wild-type and *Adamts1*-transgenic (Adamts1^Tg^) mice (*n* = 3). (**B**) Images of immunocytochemistry showing the levels and localization of MYH9 and F-actin (phalloidin) in APCs isolated from 3-week-old male wild-type mice compared with Adamts1^Tg^ littermate mice. Original magnification, ×400. (**C**) RT-qPCR quantifying *Myh9* expression levels in APCs isolated from wild-type mice and treated with recombinant ADAMTS1 (rADAMTS1) (100 ng/mL), rADAMTS1, and IWP-2 (2 μM/mL) (*n* = 3). (**D**) Immunoblots (left) with quantification (right) of MYH9 levels in APCs from wild-type mice treated with rADAMTS1 (100 ng/mL), IWP-2 (2 μM/mL), or both rADAMTS1 and IWP-2 (*n* = 4). Error bars represent mean ± SD. *P* values were calculated using *t* tests. Post hoc Bonferroni’s test was performed in **C** and **D**. **P < 0.01, ***P < 0.001.

To test whether ADAMTS1 has a role in regulating the development of adipose depots, we took advantage of the fact that a substantial portion of WAT develops during the postnatal period. Therefore, we analyzed ADAMTS1 expression levels in wild-type mice at weening, when there are high levels of adipogenesis in the gonadal adipose depot to establish the depot, compared with 8-week-old mice when the depot is largely mature. These studies revealed that expression levels of *Adamts1* mRNA were dramatically higher at 8 weeks compared with 3 weeks of age (Figure 1A). Furthermore, WAT from 8-week-old mice had substantially elevated levels of ADAMTS1 protein compared with WAT from 3-week-old mice (Figure 2B). Because the development of subcutaneous WAT depots occurs earlier than the gonadal depots (9), we measured *Adamts1* levels at DOL1 in the subcutaneous WAT and found that levels were low at this time point and increased at 3 weeks of age (Supplemental Figure 1A; supplemental material available online with this article; https://doi.org/10.1172/jci.insight.136233DS1), corresponding to the shifted developmental timing of this depot. Given what is known about the influence of ADAMTS1 on adipogenesis (8, 10), these results are consistent with a model where ADAMTS1 coordinates the reduction in adipogenesis that occurs as the WAT depot transitions from developing to homeostasis.

### MYH9 levels in APCs are regulated by ADAMTS1.

In order to reveal the impact of increased ADAMTS1 levels at 8 weeks of age on APCs, we first performed an unbiased screen for proteomic changes in APCs that occur in response to ADAMTS1. We examined the proteomes of APCs treated with recombinant ADAMTS1 (rADAMTS1) compared with treatment with control carrier protein using 2D fluorescence difference gel electrophoresis (2D-DIGE) (11). In these experiments, we elected to add recombinant ADAMTS1 to the medium of APCs in culture in order to mimic the extracellular signal we were investigating. When we examined the proteomic differences induced by ADAMTS1 treatment (Supplemental Figure 2A), we were particularly intrigued by the identification of myosin heavy chain 9 (MYH9) as a candidate target of the ADAMTS1 signal because this finding was validated by an orthogonal approach using Western blotting (Figure 1C and Supplemental Figure 2B) and because MYH9 has been connected to Wnt signaling (see below). Further, the induction of MYH9 by ADAMTS1 was blocked by the addition of the myosin 2 inhibitor blebbistatin (Bleb) (12) (Figure 1C).

MYH9 is expressed in a variety of cell types, where it is involved in processes including maintenance of cell shape and migration (13–16). This protein attracted our attention because nonmuscle myosin is connected to Wnt signaling in the setting of cell polarity and migration (17–19), and we previously identified Wnt as a mediator of the ADAMTS1 signal to APCs. To our knowledge, no connection between MYH9 and ADAMTS1 or an ADAMTS1/MYH9 pathway in regulating WAT development has been previously recognized.

We hypothesized that, if the regulation of MYH9 by ADAMTS1 was physiologically relevant, it would display expression differences in vivo in the WAT that correspond to the changes we discovered in ADAMTS1 levels across the developmental time points in wild-type mice. Indeed, we found that both *Myh9* mRNA expression and MYH9 protein levels were significantly increased in the WAT of 8-week-old mice compared with 3-week-old mice, corresponding with the increase in ADAMTS1 levels (Figure 1, A and B). Furthermore, we observed a shift in the timing of the induction of MYH9 in subcutaneous WAT depots (Supplemental Figure 1B) that synchronized to the earlier developmental timing and corresponded with the earlier induction of *Adamts1* in this depot.

To validate our findings by another approach, we compared APCs isolated from wild-type mice to APCs from *Adamts1*-transgenic (Adamts1^Tg^) mice, which overexpress *Adamts1* in adipocytes using an aP2 promoter/enhancer element. Using reverse transcription quantitative PCR (RT-qPCR), we discovered that APCs from Adamts1^Tg^ mice had increased expression of *Myh9* compared with APCs isolated from wild-type littermate controls (Figure 2A). Further, using immunocytochemistry (ICC), we confirmed that APCs isolated from Adamts1^Tg^ mice had higher levels of MYH9 compared with APCs isolated from wild-type littermate controls. We also found that a significant amount of MYH9 in Adamts1^Tg^ APCs colocalized with F-actin (Figure 2B). These results indicate that increased levels of ADAMTS1 in adipose tissue resulted in an induction of MYH9 in APCs.

### ADAMTS1 regulation of APC MYH9 occurs through Wnt signaling.

We previously elucidated that ADAMTS1 regulates Wnt signaling in APCs with induction of nuclear translocation of β-catenin (8). Nuclear translocation of β-catenin results in the modulation of the expression of Wnt target genes (20). We hypothesized that the mechanism of the induction of MYH9 in response to ADAMTS1 is by the stimulation of Wnt signaling, leading to the regulation of *Myh9* expression. Consistent with this hypothesis, we found that the small molecule Wnt inhibitor IWP-2 (21) both blocked rADAMTS1 induction of *Myh9* RNA expression (Figure 2C) and prevented ADAMTS1 induction of MYH9 protein levels (Figure 2D). These findings indicate that *Myh9* is a target gene of Wnt signaling and that ADAMTS1 induction of MYH9 results from regulation of Wnt signaling.

### Intracellular localization of ADAMTS1 induces MYH9 in APCs.

**Figure 3:**
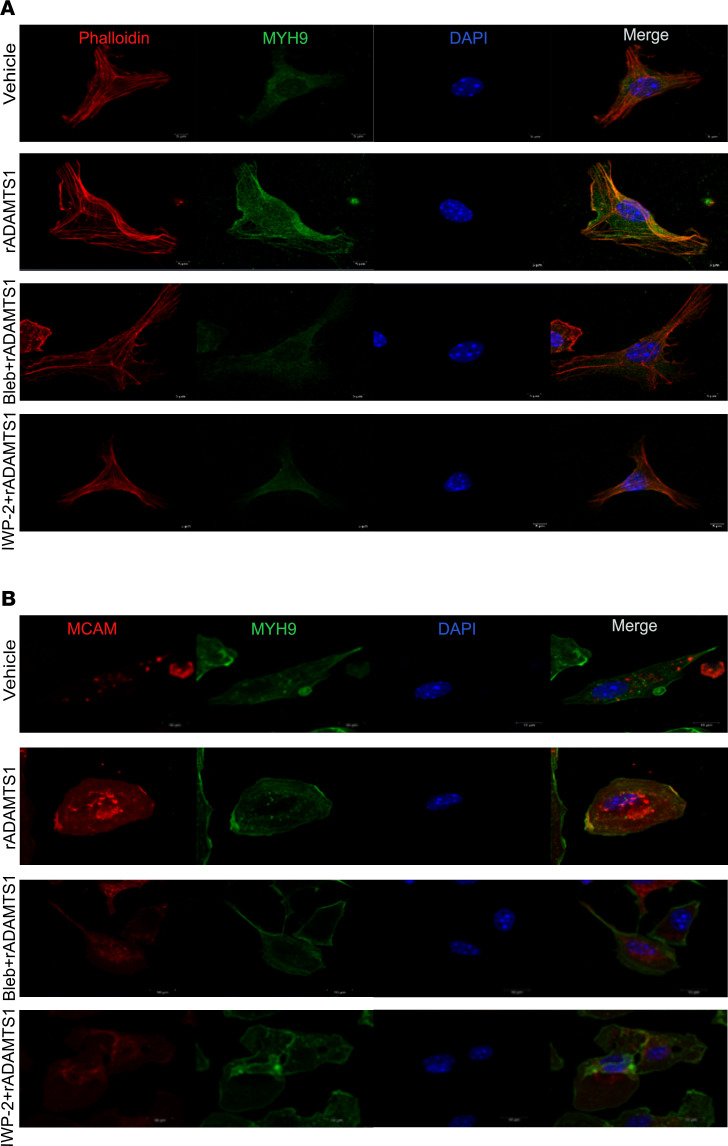
Colocalization of ADAMTS1-induced MYH9 with MCAM and F-actin. (**A**) Images of immunocytochemistry showing the localization of MYH9 and MCAM in APCs treated with rADAMTS1 (100 ng/mL) alone and rADAMTS1 in combination with Bleb (30 μM) or IWP-2 (2 μM/mL). (**B**) Images of immunocytochemistry showing the localization of MYH9 and F-actin (phalloidin) in APCs treated with rADAMTS1 (100 ng/mL) alone and rADAMTS1 in combination with Bleb (30 μM) or IWP-2 (2 μM/mL). Original magnification, ×400.

MYH9 is a part of a protein network that includes melanoma cell adhesion molecule (MCAM, also known as CD146 and MUC18) that can also recruit F-actin and induce localized increases in cytoplasmic calcium levels (18). Therefore, we performed ICC experiments to elucidate the intracellular locations of these proteins in APCs. These studies revealed that exposing APCs to rADAMTS1 induced MYH9 and enhanced a colocalization of MYH9 with F-actin (Figure 3A) and MCAM (Figure 3B). The addition of Bleb or IWP-2 severely attenuated these ADAMTS1-driven effects (Figure 3, A and B).

### ADAMTS1 regulation of MYH9 gates adipogenesis ex vivo and in vivo.

**Figure 4:**
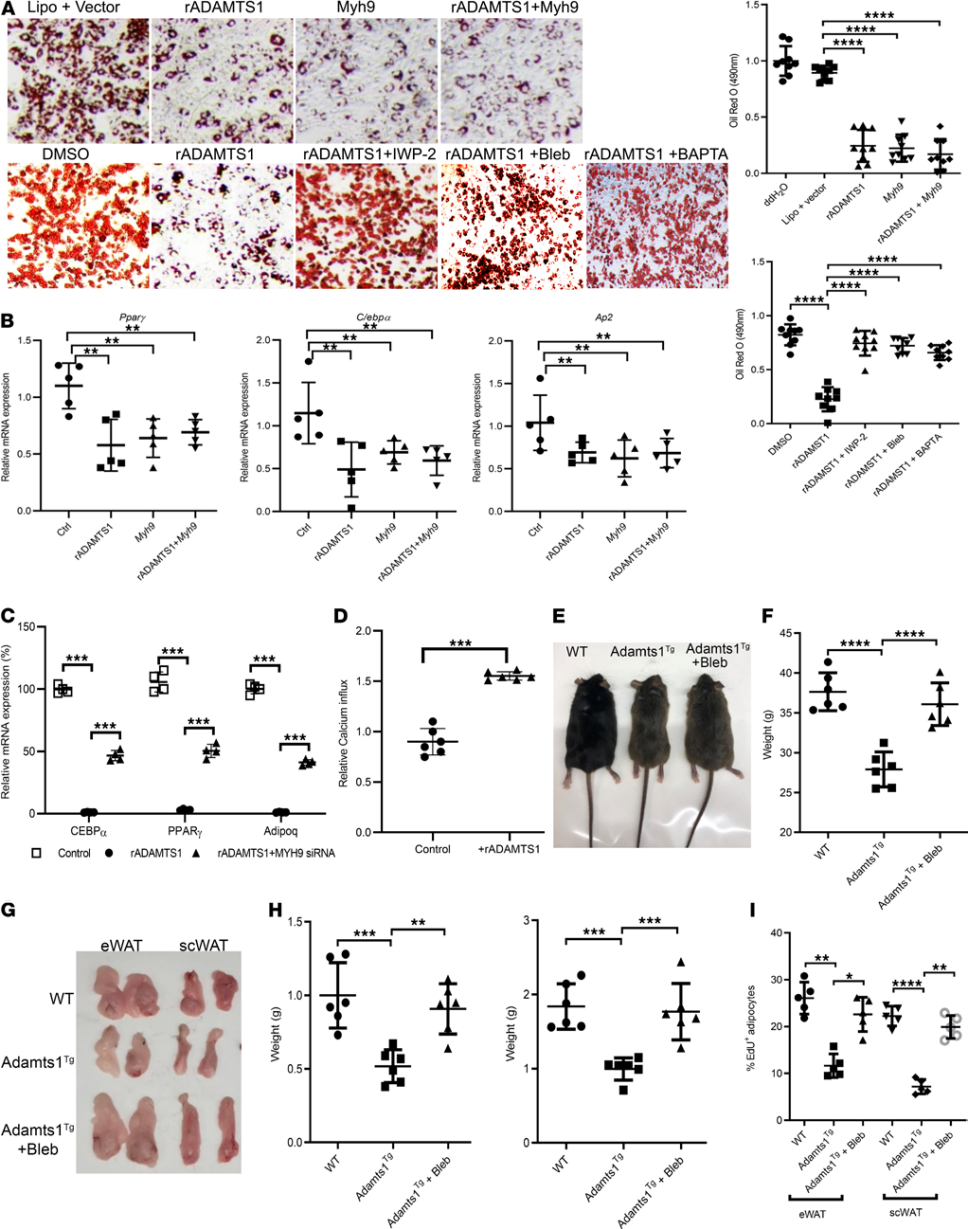
Adamts1/Wnt/Myh9 pathway regulates adipogenesis. (**A**) (Left) Representative images from light-phase microscopy of APCs isolated from wild-type mice 8 days after the induction of adipogenesis (with dexamethasone, insulin, and 3-Isobutyl-1-methylxanthine) and stained with oil red O and (right) quantification of oil red O levels using spectroscopy. Original magnification, ×100. (**B**) RT-qPCR quantifying the expression levels of markers of adipogenesis (*Pparγ*, *C/ebpα*, and *Ap2*) in APCs treated with rADAMTS1 or *Myh9* or both (*n* = 5). (**C**) RT-qPCR monitoring the expression levels of markers of adipogenesis (*Pparγ*, *C/ebpα*, and *Adipoq*) after treatment with rADAMTS1 or rADAMTS1 rescued with *Myh9* siRNA (*n* = 4). (**D**) Measurements of the calcium influx stimulated by rADAMTS1 exposure (*n* = 6). (**E**) Images of male wild-type, *Adamts1*-transgenic (Adamts1^Tg^), and Adamts1^Tg^ mice treated with Bleb (Adamts1^Tg^ + Bleb) mice. (**F**) Quantification of the whole-body weights of wild-type, Adamts1^Tg^, and Adamts1^Tg^ + Bleb mice (*n* = 6). (**G**) Representative images of gross dissected epididymal (eWAT) and subcutaneous (scWAT) white adipose tissue depots from wild-type, Adamts1^Tg^, and Adamts1^Tg^ + Bleb mice. (**H**) Quantification of the wet weights of scWAT and eWAT of wild-type, Adamts1^Tg^, and Adamts1^Tg^ + Bleb mice (*n* = 6 mice for each type). (**I**) Quantification of the percentage of EdU^+^ adipocytes in eWAT and scWAT of wild-type, Adamts1^Tg^, and Adamts1^Tg^ + Bleb mice by cytometry. Error bars represent mean ± SD. *P* values were calculated using *t* test (4D) or 1-way ANOVA with Tukey’s multiple comparisons post hoc test (**A**–**C**, **F**, **H**, and **I**). *P < 0.05, **P < 0.01, ***P < 0.001, ****P < 0.0001.

We next tested whether the Adamts1/Myh9 pathway we identified is relevant for adipogenesis. As anticipated, we found that exposing APCs to rADAMTS1 inhibited adipogenesis, measured by both oil red O staining (which stains neutral lipids) (Figure 4A) and quantification of the expression level of adipogenesis markers (Figure 4B and Supplemental Figure 3). We discovered that inhibiting Wnt signaling in APCs with IWP-2 or, importantly, blocking myosin 2 with Bleb, was sufficient to rescue adipogenesis from the ADAMTS1 inhibition of adipogenesis (Figure 4A and Supplemental Figure 3). Remarkably, overexpression of *Myh9* was sufficient to inhibit adipogenesis to similar levels as exposure to rADAMTS1 (Figure 4, A and B), and there was no additive activity to the inhibition of adipogenesis with combined ADAMTS1 exposure and *Myh9* overexpression (Figure 4, A and B). Next, we tested whether knocking down *Myh9* would rescue APCs from the ADAMTS1-induced block in adipogenesis. Using siRNA, we were able to reduce MYH9 levels in APCs by approximately 60% (Supplemental Figure 4), which resulted in approximately 50% rescue of the ADAMTS1 block in adipogenesis (Figure 4C). Together, these findings established that the Adamts1/Myh9 pathway regulates adipogenesis in the context of induced differentiation in cell culture assays.

Wnt signaling converges on nonmuscle myosin and MCAM proteins to regulate intracellular calcium concentrations (22). To test whether ADAMTS1 regulates intracellular calcium concentrations in APCs, we exposed APCs to rADAMTS1 and monitored the intracellular calcium levels. These studies confirmed that exposure to extracellular ADAMTS1 modulated intracellular calcium levels in APCs (Figure 4D). This was intriguing to us because intracellular calcium is critical for gating the initiation of adipogenesis (23). Therefore, we hypothesized that this was a mechanism by which the Adamts1/Wnt/Myh9 pathway regulates adipogenesis. To test this hypothesis, we monitored the effect of 1,2-bis(o-aminophenoxy)ethane-N,N,N′,N′-tetraacetic acid (BABTA), which is a highly selective membrane permeant chelator of intracellular calcium (24), on ADAMTS1 activity. We discovered that the addition of BABTA was sufficient to rescue APCs from ADAMTS1 inhibition of adipogenesis as monitored by oil red O staining (Figure 4A) and quantified by measuring the expression levels of markers of adipogenesis (Supplemental Figure 3). These findings implicate the regulation of intracellular calcium as a critical mechanism by which the Adamts1/Myh9 pathway modulates adipogenesis.

To test our findings in vivo, we administered the myosin 2 inhibitor Bleb to Adamts1^Tg^ mice and littermate controls starting at 3 weeks of age and continuing until the mice reached 8 weeks. We confirmed that Adamts1^Tg^ were lean (Figure 4, E and F), with considerably decreased development of mature adipose tissue compared with wild-type littermate controls (Figure 4, G and H). Importantly, we discovered that treatment with Bleb was sufficient to rescue this phenotype in vivo, restoring the development of adipose tissue back to similar levels as occurred in the littermate control mice (Figure 4, G and H). Further, histological analysis of the adipose tissue showed that the adipocytes in Adamts1^Tg^ treated with Bleb were similar in size to untreated mice (Supplemental Figure 5), suggesting that the rescue of the phenotype was due to increased adipogenesis.

Finally, we directly tested the role of Adamts1 regulation of myosin in gating adipogenesis in vivo during depot development. To measure the level of adipogenesis occurring in vivo, we employed a 5-ethynyl-2′-deoxyuridine (EdU) pulse-chase technique that we previously developed and described in detail (8, 10, 25). Briefly, in this method, APCs are labeled by injecting EdU into young mice when there are high levels of proliferation in the adipose depots. After a chase period, mature adipocytes are isolated and purified, and the percentage of EdU-positive mature adipocytes of the total adipocytes in the depot are quantified by cytometry. Because mature adipocytes do not divide, the EdU-positive adipocytes must arise from APCs that were labeled during the pulse period and differentiated during the chase period. As anticipated, we found that Adamts1^Tg^ mice had lower levels of adipogenesis during adipose depot development compared with control littermates (Figure 4I). Also as expected, because Adamts1 levels were low during adipose depot formation, treatment of wild-type control mice with Bleb did not alter adipogenesis. Strikingly, treating Adamts1^Tg^ mice with Bleb was sufficient to remove this block on adipogenesis (Figure 4I). These findings indicate that regulation of myosin was sufficient to gate adipogenesis during adipose depot development in vivo.

## Discussion

Adipose tissue has an integral role in physiology, with both too much (obesity) and too little (lipodystrophy) leading to pathology. Given the importance of adipocytes for maintaining health, significant efforts have been made to elucidate the pathways that regulate the process of adipocyte formation. As a result of this extensive research by many laboratories, a detailed map has emerged of cell-intrinsic pathways that regulate adipogenesis. However, inherently, these approaches exclude physiological inputs that signal to APCs. As a result, much less is known about how adipose tissue depots develop or how extracellular signals regulate this process in vivo.

Our studies revealed a previously unrecognized pathway where an extracellular signal converges on a major cytoskeletal protein, MYH9, in APCs to regulate adipogenesis during depot formation in vivo. Our identification of the cytoskeletal protein MYH9 as a downstream target of the Adamts1 signal in APCs is highly interesting. It is understood that APCs must undergo a substantial change in cell size and shape in order to become mature, large, lipid-laden adipocytes (26). However, the results of our studies identifying and examining a direct pathway from an extracellular signal to the regulation of MYH9 indicate that this regulation does not only facilitate the change in cell size but also serves as a potent gate on the differentiation process itself.

Within this same consideration, Wnt signaling is a critical factor directing developmental and differentiation decisions in a number of contexts (27). Our studies detail a mechanism for Wnt signaling to regulate differentiation that is transmitted by cytoskeletal proteins, which is beyond the recognized role of Wnt signaling to cytoskeletal proteins that regulates cell migration (17); further, our results elucidate that this newly identified Adamts1/Wnt/Myh9 pathway is a major regulator of adipogenesis during adipose depot formation. Given the broad influence of Wnt signaling in developmental and stem cell biology, it will be of considerable interest to investigate whether the convergence of Wnt signaling on cytoskeletal proteins gates stem cell differentiation and tissue development in other contexts as well.

## Methods

### Mice

The generation of the *Adamts1*-transgenic line was previously described and performed in-house (8). Mice were housed under 12-hour light/12-hour dark cycles.

### Isolation of stromal vascular fraction and APCs

Subcutaneous and gonadal adipose depots were dissected from the animals, minced, and incubated in DMEM/F12 containing 1 mg/mL of collagenase (Worthington) for 1 hour at 37°C with 5% CO_2_. The collagenase was then inactivated by the addition of an equal volume of DMEM containing 10% FBS. The samples were subjected to gentle trituration with a 25 mL pipette and then passed through a 100 μm cell strainer. Samples were then centrifuged and the cells were resuspended in 1 mL of ACK lysing buffer (Lonza) to remove red blood cells. After inactivation with HBSS with 2% FBS, the adipose tissue stromal cells were either plated for use in experiments or labeled for isolation of APCs. For isolation of APCs, stromal vascular fraction was labeled using the following conjugated antibodies: SCA1 FITC (BD Biosciences, 557405), CD45 phycoerythrin–Cy7 (eBioscience, Thermo Fisher Scientific, 25-0421-82), and CD31 APC (eBioscience, Thermo Fisher Scientific, 17-0311-82). Samples were sorted in a FACS core facility. Cell gating was based on comparison with unstained and fluorescence minus one–stained controls. Single live cells were discriminated by forward-scatter and side-scatter analyses and 7-aminoactinomycin D labeling (BD Biosciences). Cells were sorted into serum-free DMEM for gene expression analysis or into complete medium (DMEM with 10% FBS [Atlanta Biologicals] and streptomycin [50 μg/mL] and penicillin [50 U/mL] [Lonza]) for cell culture. Flow cytometry standard files were analyzed using FlowJo version 10.

### 2D-DIGE

#### Protein extraction, labeling, and electrophoresis.

Adipocyte progenitor cells were grown to confluence and exposed to 100 ng/mL recombinant ADAMTS1 (R&D Systems, Bio-Techne) or carrier (BSA) control protein for 3 hours and lysed in RIPA protein sample buffer. Protein was precipitated and resuspended in 2D-DIGE compatible buffer (30 mM Tris-HCl, pH 8.8, containing 7 M urea, 2 M thiourea, and 4% CHAPS). For each sample, 50 μg of protein was labeled with CyDye and equal amounts of treated and control samples were mixed together. After mixing, samples were loaded into immobilized pH gradient (IPG) gels and focused using an IPGPhor instrument (GE Healthcare, now Cytiva) and then transferred to a Criterion precast gel (Bio-Rad) for electrophoresis. Gels were scanned using an Ettan DIGE Imager (Cytiva) to detect respective CyDye-labeled proteins. DeCyder software was used for analysis and quantification of the protein spots.

#### Spot isolation and digestion.

Gel spots with differential levels between treated and control samples were cut from the gel in 1 mm^3^ cubes and placed in microcentrifuge tubes. The cubes were washed with 100 mM ammonium bicarbonate/acetonitrile (1:1, v/v) solution and then incubated with acetonitrile for 10 minutes. The gel pieces were then rehydrated in 10 mM DTT and 100 mM ammonium bicarbonate at 56°C for 1 hour. The supernatant was removed, and the gel pieces were incubated in fresh acetonitrile for 10 minutes followed by an incubation in 50 mM iodoacetamide and 100 mM ammonium bicarbonate in the dark at room temperature for 1 hour followed by another incubation with acetonitrile for 10 minutes. After removing the acetonitrile, the gel pieces were placed in trypsin protease solution for in-gel digestion and then incubated in ammonium bicarbonate/acetonitrile solution (1:2, v/v) at 37°C for 1 hour. The extracted peptides were lyophilized to near dryness and resuspended in 0.1% formic acid for liquid chromatography–mass spectrometry (LC-MS/MS) analysis.

#### Protein identification.

Samples were run on an Easy nLC1000 (Thermo Fisher Scientific) using 100 μm × 10 cm columns packed with a reversed-phase ReproSil-Pur C18-AQ resin at 600 nL/min flow rate and 5%–30% acetonitrile followed by mass spectroscopy (MS) using Q Exactive (Thermo Fisher Scientific) with a 2.2 kV spray voltage at 270°C. The MS parameters were as follows: MS resolution 60,000 at 400 *m/z;* MS precursor *m/z* range 300.0–1650.0. The MS/MS parameters were as follows: product ion scan range — start from *m/z* 100; activation type — CID; minimum signal required — 1500.0; isolation width — 3.00; normalized collision energy — 40.0; default charge state — 6; activation Q — 0.250; activation time — 30.000. For data-dependent MS/MS, the parameter was the top 5 most intense peptide ions from the preview scan in the Orbitrap.

Raw MS files were analyzed and searched against a mouse protein database using Maxquant (1.5.6.5). The parameters were set as follows: the protein modifications were carbamidomethylation (C) (fixed); oxidation (M) (variable); enzyme specificity — trypsin; maximum missed cleavages — 2; precursor ion mass tolerance — 10 ppm; MS/MS tolerance — 0.6 Da.

### Adipogenesis assays

APCs were purified from adipose tissue by FACS as described above and seeded into tissue culture plates. APCs were then grown to confluence at 37°C in 5% CO_2_. Cells were maintained at confluence for 2 days to enable exit from the cell cycle and then induced with a differentiation cocktail (1 μM dexamethasone, insulin [1 μg/mL], and 0.5 mM 3-isobutyl-1-methylxanthine) for 2 days. Thereafter, cells were maintained in maintenance medium (culture medium with insulin [1 mg/mL]), which was changed every other day. Differentiation was allowed to continue for a total of 8 days.

### Immunoblots

Cells were lysed using SDS lysis buffer (50 mM tris-HCl at pH 6.8, 2% SDS, 10 mM dithiothreitol, and 10% glycerol). Total protein was quantified using Bradford assays (Thermo Fisher Scientific). The proteins were separated by electrophoresis in 10% SDS polyacrylamide gels and transferred to PVDF membranes. Membranes were probed with primary antibodies (ADAMTS1; 1:1000; R&D Systems, Bio-Techne; catalog AF5867), MYH9 (1:1000; Cell Signaling Technology, catalog 3403), β-actin (1:2000; GenScript, catalog A007002) and subsequently with secondary antibodies conjugated with HRP (Santa Cruz Biotechnology catalog 2004 and 2473) followed by enhanced chemiluminescence (Cytiva) and digital imaging using a ChemiDoc imaging system (Bio-Rad).

### Myh9 knockdown

Preadipocytes were isolated from wild-type mice, grown to approximately 80% confluence, and transfected with 0.3 μM Myh9 siRNA (Santa Cruz Biotechnology) or scramble control siRNA (Santa Cruz Biotechnology) using Lipofectamine 2000 (Thermo Fisher Scientific) and then grown to confluence for 2 days. The knockdown efficiency was measured by immunoblotting after cells reached confluence for 2 days.

### RT-qPCR

Total RNA was extracted from cells and tissue using RNeasy kits (QIAGEN) according to the manufacturer’s protocol. Equal amounts of RNA were reverse-transcribed using random priming. The relative transcript levels were quantified and normalized to the expression of housekeeping genes using the SYBR Green method in a CFX384 detection system cycler (Bio-Rad) and 2^–ΔΔCt^ values were calculated.

### In vivo EdU pulse-chase

EdU (Carbosynth) was administered by i.p. injections (100 mg/kg) once a day for 2 weeks. Mice were euthanized by asphyxiation with CO_2_ and cervical dislocation. Subcutaneous and gonadal adipose depots were dissected from the animals, minced, and incubated in DMEM/F12 containing 1 mg/mL of collagenase (Worthington) for 1 hour at 37°C with 5% CO_2_. The collagenase was then inactivated by the addition of an equal volume of DMEM containing 10% FBS. The samples were subjected to gentle trituration with a 25 mL pipette. The digested tissue suspension was filtered through a 200 μm mesh and then centrifuged at 50*g* for 5 minutes. Mature adipocytes were removed from the upper part of the solution and placed in DMEM/F12 followed by a wash and recentrifugation. Adipocyte nuclei were isolated in cold nuclei extraction buffer (320 mM sucrose, 5 mM MgCl_2_, 10 mM Hepes, and 2% Triton X-100 at pH 7.4) using a Dounce homogenizer. Nuclei were centrifuged at 3000*g* for 15 minutes and washed with a cold washing buffer (320 mM sucrose, 5 mM MgCl_2_, 10 mM HEPES, 1% BSA, and 0.1% sodium azide at pH 7.4). EdU nuclei were labeled using the Click-iT EdU Alexa Fluor 488 Flow Cytometry Assay Kit (Invitrogen, Thermo Fisher Scientific). DAPI (500 ng/mL) was added immediately before FACS analysis. EdU gating was based on samples isolated from mice that had not been injected with EdU but processed in parallel.

### Calcium measurement

The intracellular calcium levels were monitored using Fluo 3-AM (Thermo Fisher Scientific). APCs were cultured in 98-well plates and treated with 1 mM Fluo 3-AM and 20% Pluronic F-127 (MilliporeSigma) followed by incubation for 30 minutes at room temperature. After washing, baseline fluorescence was quantified in a plate reader (Tecan) at excitation 485 nm and emission at 538 nm. APCs were then treated with 400 ng/μL of rADAMTS1 (R&D Systems, Bio-Techne) or control BSA for 2 minutes, and fluorescence intensity was quantified again in the plate reader.

### Bleb treatment

Three-week-old male Adamts1^Tg^ and littermate control mice were treated with 2 mg/kg Bleb (Selleckchem) or carrier control (2% DMSO, 40% PEG-300, and double-distilled H_2_O) by daily i.p. injection until 8 weeks of age.

### Statistics

Statistical analysis was performed by 1- or 2-way ANOVA using GraphPad Prism software. Differences between experimental groups were determined by 2-tailed *t* test. *P* values less than 0.05 were considered significant.

### Study approval

All studies using mice were approved by the IACUCs at Stanford University and the University of California, San Francisco (UCSF).

## Author contributions

BJF conceived the project and experiments, analyzed results, and wrote the manuscript with input from all authors. SYC, MS, KN, and LL conducted experiments, analyzed results, and assisted with the generation of the figures and the writing of the manuscript. SYC is listed first in the author list to correspond with the timing of who started to work on the project first.

## Supplementary Material

Supplemental data

## Version 1 05/10/2021

Electronic publication
