# Supplementary material for: MYH9 facilitates autoregulation of adipose tissue depot development
Source: JCI Insight. 2021 May 10;6(9):e136233. doi: 10.1172/jci.insight.136233 (PMC8262332; doi:10.1172/jci.insight.136233)
Supplement: Supplemental data [file jciinsight-6-136233-s101.pdf]

Supplemental Figure 1

A

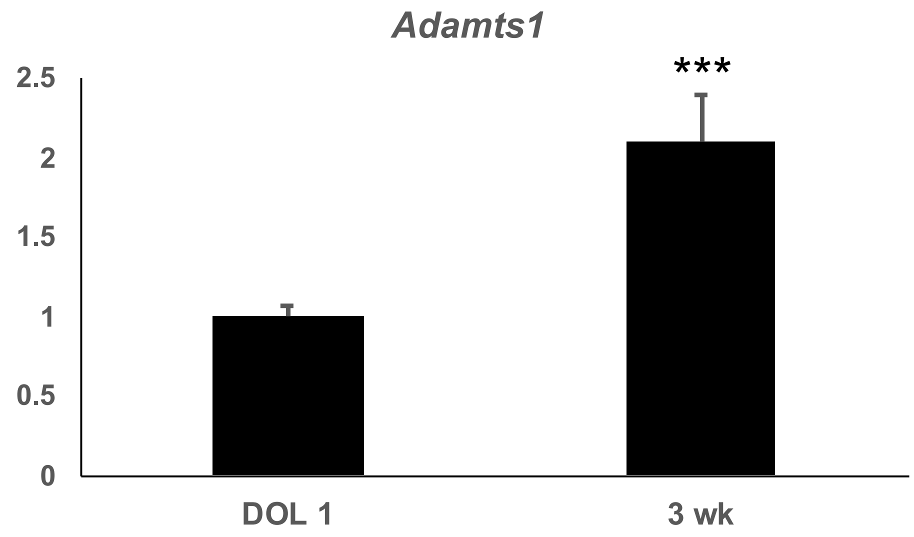

B

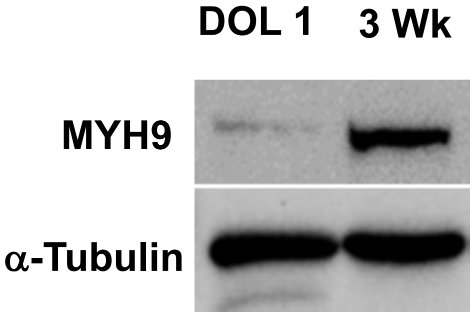

Supplemental Figure 1: Expression Levels of Adamts1 and Myh9 in Subcutaneous Adipose Depots

(A)RT-qPCR measuring the relative levels of *Adamts1* expression at DOL1 and 3 weeks of age. Adipose depots at DOL1 were too small to obtain sufficient protein to specifically detect ADAMTS1 by immunoblotting. **(B)** Immunoblots comparing MYH9 levels at DOL1 and 3 weeks of age. N=3. Error bars represent ±SD from the mean. P values were calculated using t-tests *** P<0.001

Supplemental Figure 2

A

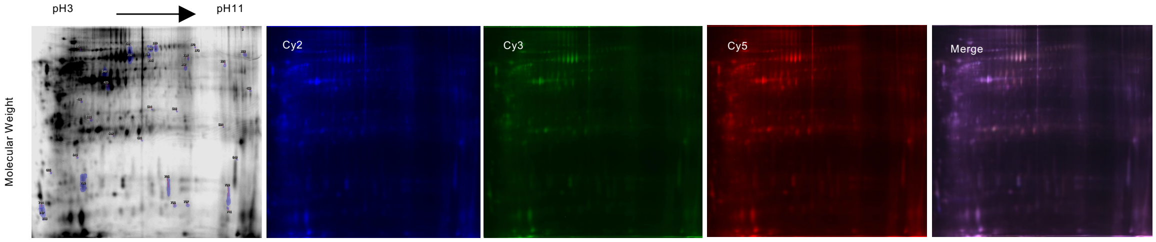

Top upregulated hits		Top downregulated hits	
Protein name	Gene	Protein name	Gene
cAMP-dependent protein kinase type I-alpha regulatory subunit	Prkar1a	TRIO and F-actin-binding protein	Triobp
Nuclear migration protein nudC	Nudc	WD repeat-containing protein 1	Wdr1
Protein disulfide-isomerase	P4hb	Cleavage stimulation factor subunit 2	Cstf2
Succinyl-CoA ligase [ADP-forming] subunit beta, mitochondrial	Sucla2	BAG family molecular chaperone regulator 3	Bag3
Thioredoxin domain-containing protein 5	Txndc5	Transgelin-2	Tagln2
Homer protein homolog 3	Homer3	Destrin	Dstn
Drebrin-like protein	Dbnl	Chloride intracellular channel protein 1	Clic1
Spectrin alpha chain, non-erythrocytic 1	Sptan1	Astrocytic phosphoprotein PEA-15	Pea15
Myosin-9	Myh9	COP9 signalosome complex subunit 4	Cops4
ATP synthase subunit beta, mitochondrial	Atp5b	NudC domain-containing protein 2	Nudcd2

KEGG Pathway analysis: Upregulated pathways						
Rank	Term	Count	p-value	Fold Enrichment	Benjamini	FDR
1	Citrate cycle (TCA cycle)	9	6.70E-06	8.4	8.00E-04	7.70E-03
2	Glycolysis/Gluconeogenesis	12	1.60E-05	5.1	9.80E-04	1.90E-02
3	Gap junction	13	3.10E-05	4.4	1.30E-03	3.60E-02
4	Proteosome	8	9.90E-04	4.9	2.30E-02	1.10E+00
5	Spliceosome	13	1.00E-03	3	2.10E-02	1.20E+00

KEGG Pathway analysis: Downregulated pathways						
Rank	Term	Count	p-value	Fold Enrichment	Benjamini	FDR
1	Spliceosome	12	2.50E-05	4.9	2.10E-03	2.70E-02
2	Glycolysis/Gluconeogenesis	9	4.60E-05	6.7	1.90E-03	4.90E-02
3	Tight junction	12	5.60E-05	4.5	1.50E-03	6.00E-02
4	Antigen processing and presentation	8	1.90E-03	4.5	3.00E-02	2.00E+00
5	Regulation of actin cytoskeleton	12	3.20E-03	2.8	4.30E-02	3.40E+00

Database of Annotation, Visualization, and Integrated Discovery (DAVID) Analysis v6.7

Rank	Category	Cluster Term	Count	Enrichment Score	p-value	Fold Change	Benjamini	FDR
1	GOTERM_CC_FAT	Cytoskeleton	106	27.02	2.10E-32	3.5	6.80E-30	2.80E-29
	GOTERM_CC_FAT	Cytoskeletal part	80		2.40E-26	3.9	4.00E-24	3.30E-23
2	GOTERM_MF_FAT	Actin binding	38	14.05	7.40E-21	7.1	6.10E-19	1.00E-17
	GOTERM_BP_FAT	actin filament-based process	28		7.80E-14	6.1	3.20E-11	1.30E-10
	GOTERM_BP_FAT	actin cytoskeleton organization	27		1.20E-13	6.3	3.90E-11	2.00E-10
	GOTERM_CC_FAT	actin cytoskeleton	30		1.50E-13	5.5	5.90E-12	2.00E-10
3	GOTERM_MF_FAT	Nucleotide-binding	141	11.62	6.90E-22	2.2	3.10E-19	9.70E-19
	GOTERM_MF_FAT	Purine ribonucleotide binding	107		2.00E-13	2	1.50E-11	2.80E-10
	GOTERM_MF_FAT	ATP binding	83		1.50E-09	2	5.60E-08	2.10E-06
4	GOTERM_CC_FAT	Intermediate filament	22	10.91	8.00E-11	6	2.60E-09	1.10E-07
	GOTERM_CC_FAT	Intermediate filament cytoskeleton	22		1.20E-10	5.9	3.60E-09	1.60E-07
5	GOTERM_CC_FAT	Contractile Fiber	22	9.87	4.70E-14	8.7	3.00E-12	6.30E-11
	GOTERM_CC_FAT	Myofibril	20		2.30E-12	8.2	8.30E-11	3.10E-09
6	GOTERM_BP_FAT	Cellular macromolar complex subunit organization	35	8.61	8.80E-16	5.5	1.40E-12	1.50E-12
	GOTERM_MF_FAT	GTPase Activity	23		1.60E-11	6.2	1.00E-09	2.30E-08
	GOTERM_BP_FAT	Microtubule-based movement	17		6.30E-09	6.5	7.40E-07	1.10E-05
	GOTERM_BP_FAT	protein complex biogenesis/assembly	21		1.90E-06	3.6	1.10E-04	3.10E-03
	KEGG_PATHWAY	Gap Junction	13		1.70E-04	3.7	3.60E-03	2.00E-01
7	GOTERM_CC_FAT	Vesicle	33	7.65	8.55E-06	2.4	9.60E-05	1.20E-05
	GOTERM_CC_FAT	cytoplasmic membrane-bounded vesicle	28		1.70E-04	2.5	1.00E-04	2.20E-02
8	GOTERM_BP_FAT	mRNA processing	27	7.37	4.50E-09	4	5.60E-07	7.50E-06
	GOTERM_MF_FAT	RNA binding	47		5.50E-08	2.4	1.80E-06	7.80E-05
	GOTERM_BP_FAT	mRNA metabolic process	27		8.40E-08	3.4	7.60E-06	1.40E-04
	GOTERM_BP_FAT	RNA splicing	20		1.20E-06	3.8	7.70E-05	2.00E-03
9	GOTERM_CC_FAT	Cell cortex	20	7.22	8.70E-10	5.9	2.20E-08	1.20E-06
10	GOTERM_BP_FAT	glucose metabolic process	20	6.16	3.50E-09	5.5	5.10E-07	5.80E-06
	GOTERM_BP_FAT	generation of precursor metabolites and energy	27		4.10E-09	4	5.60E-07	6.90E-06
	GOTERM_BP_FAT	glycolysis	11		1.40E-07	9.6	1.10E-05	2.30E-04
	KEGG_PATHWAY	Glycolysis/Gluconeogenesis	13		1.60E-05	4.6	6.60E-04	1.80E-02
	KEGG_PATHWAY	Pyruvate metabolism	7		6.00E-03	4.2	5.60E-02	6.70E+00

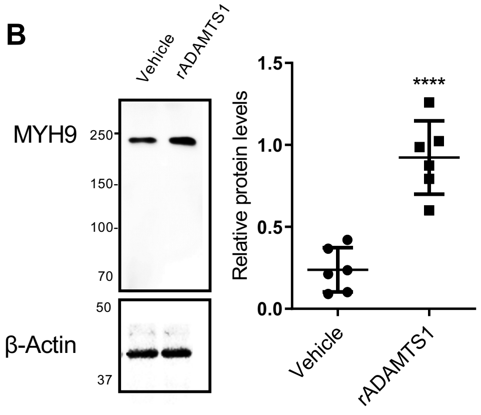

Supplemental Figure 2: Proteomic Analysis of the Response to rADAMTS1

(A) Whole cell proteome analysis of preadipocytes treated with recombinant ADAMTS1 compared to vehicle treatment control using two-dimensional fluorescence difference gel electrophoresis (2D-DIGE). **(B)** Immunoblot (left) and quantification (right) of the induction of MYH9 in response to rADAMTS1 (n=6).

Error bars represent ±SD from the mean. P values were calculated using t-tests **** P<0.0001

Supplemental Figure 3

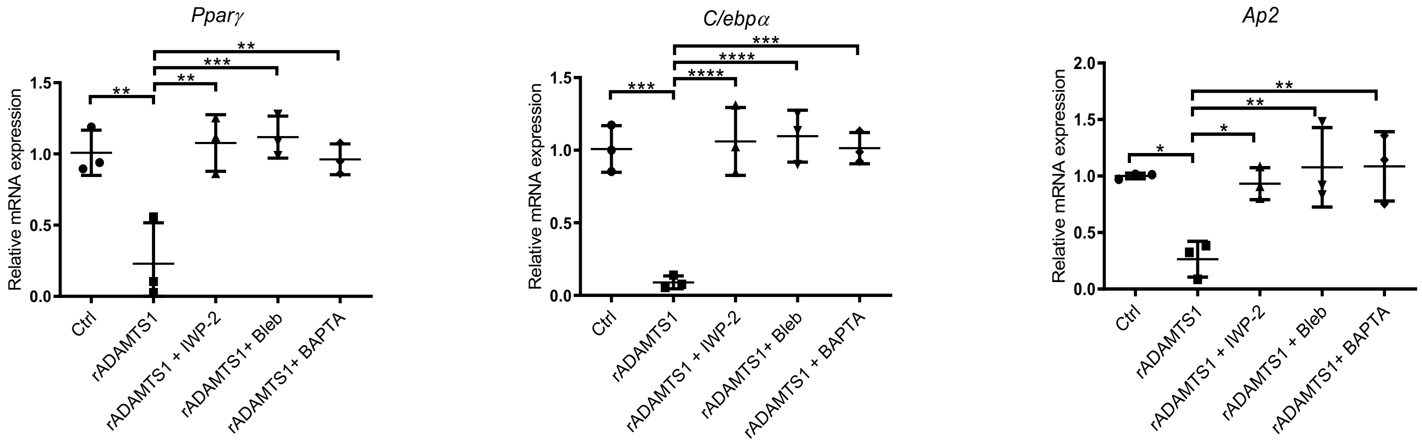

Supplemental Figure 3: Adamts1-Wnt-Myh9 Pathway Regulates Adipogenesis
RT-qPCR quantifying the expression levels of markers of adipogenesis in APCs treated with rADAMTS1 alone and with IWP-2, Bleb or BAPTA (n=3).
Error bars represent ±SD from the mean. P values were calculated using t-tests followed by Bonferroni corrections. *p<0.05, **p<0.01, ***p<0.001, ****p<0.0001.

Supplemental Figure 4

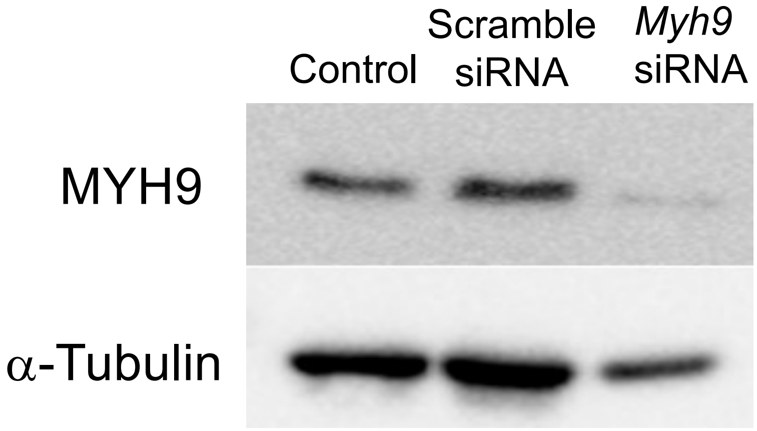

Supplemental Figure 4: Validation of Myh9 knockdown using siRNA: Immunoblots from preadipocyte cell lysates harvested 48 hrs after being transfected with either scramble or *Myh9* specific siRNA, demonstrating that *Myh9* specific siRNA results in ~60% knockdown in MYH9 levels.

Supplemental Figure 5

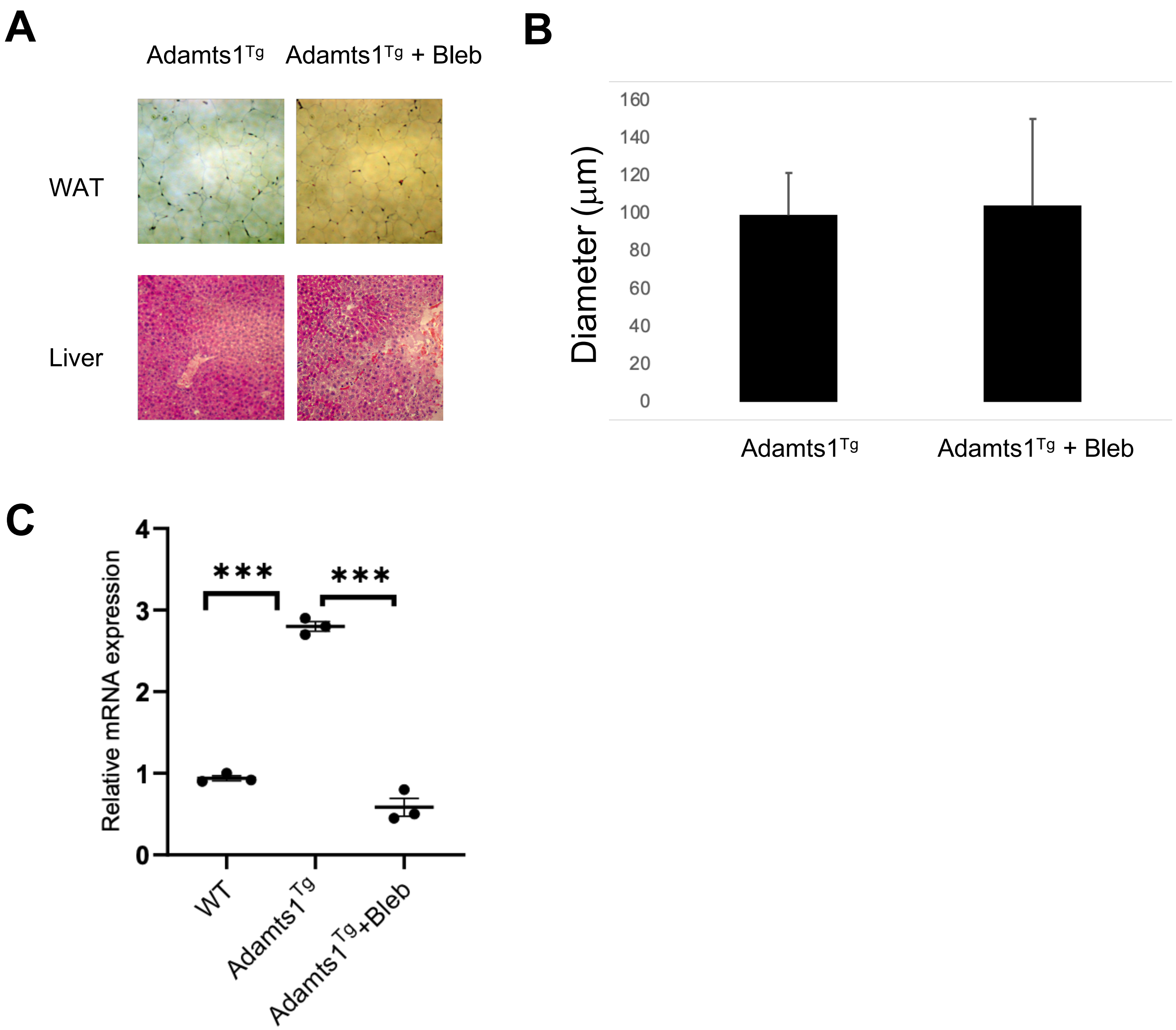

Supplemental Figure 5: Bleb treatment of Adamts1^{Tg} mice:
(A) Images of histological sections from WAT and liver. **(B)** Quantification of adipocyte size (μm) using ImageJ analysis of the histological sections. There is no statistical difference in the adipocyte size. **(C)** RT-qPCR quantifying the expression level of *Myh9* in WAT (n=3)
Error bars represent ±SD from the mean. P values were calculated using t-tests followed by Bonferroni correction. *** $P<0.001$
